# KDM2A-dependent reduction of rRNA transcription on glucose starvation requires HP1 in cells, including triple-negative breast cancer cells

**DOI:** 10.18632/oncotarget.27092

**Published:** 2019-07-30

**Authors:** Kengo Okamoto, Yuji Tanaka, Sachiko Ogasawara, Chikashi Obuse, Jun-ichi Nakayama, Hirohisa Yano, Makoto Tsuneoka

**Affiliations:** ^1^ Laboratory of Molecular and Cellular Biology, Faculty of Pharmacy, Takasaki University of Health and Welfare, Takasaki, Japan; ^2^ Department of Pathology, Kurume University School of Medicine, Kurume, Japan; ^3^ Department of Biological Sciences, Graduate School of Science, Osaka University, Osaka Japan; ^4^ Division of Chromatin Regulation, National Institute for Basic Biology, Okazaki, Japan

**Keywords:** HP1, KDM2A, nucleoli, rRNA, TNBC

## Abstract

Triple-negative breast cancer (TNBC) is very aggressive and lacks specific therapeutic targets. Ribosome RNAs (rRNAs) are central components of ribosomes and transcribed in nucleoli, and the level of rRNA transcription greatly affects ribosome production and cell proliferation. We have reported that an epigenetic protein, KDM2A, exists in nucleoli and reduces rRNA transcription on glucose starvation. However, the molecular mechanism is still unclear. The purpose of this study is to examine the KDM2A-dependent regulation mechanism of rRNA transcription. In this study, we turned our attention to the nucleolar accumulation of KDM2A. We found that KDM2A had multiple regions for its nucleolar localization, and one of the regions was directly bound by heterochromatin protein 1γ (HP1γ) using valine 801 in the LxVxL motif of KDM2A. A knockdown of HP1γ or a point mutation of valine 801 in KDM2A decreased the nucleolar accumulation of KDM2A, and suppressed the reduction of rRNA transcription on glucose starvation. These results uncovered a novel function of HP1γ: the regulation of rRNA transcription, and suggested that HP1γ stimulates the nucleolar accumulation of KDM2A to support the KDM2A-dependent regulation of rRNA transcription. HP1γ was expressed in cancer cells in all breast carcinoma tissues examined, including TNBC tissues. A knockdown of HP1γ in a TNBC cell line, MDA-MB-231 cells, reduced the nucleolar accumulation of KDM2A, and suppressed the reductions of rRNA transcription and cell proliferation on glucose starvation. These results suggest that the KDM2A-dependent regulation of rRNA transcription requires HP1γ, and thus may be applicable to the treatment of TNBC.

## INTRODUCTION

Among women, breast cancer is the most commonly diagnosed cancer and the leading cause of cancer death worldwide [1, 2]. Tumors that do not express estrogen receptors (ER), progesterone receptors (PR), and do not have HER2/Neu amplification are referred to as triple-negative breast cancers (TNBCs) [3]. TNBCs represent approximately 10 to 15% of all breast cancers, and patients with TNBC have a poor prognosis compared to the other subtypes of breast cancer [3–5]. Although improved understanding of breast cancer has allowed the development of more effective treatments, no targeted drug has been approved for treatment of TNBC [6, 7]. Identification of the mechanism regulating cell proliferation of TNBC could be applied to the treatment of the cancers.

Ribosome synthesis largely affects cell growth, and the rate of ribosome synthesis is tightly regulated in mammalian cells [8]. Ribosomal RNAs (rRNAs) are central components of the ribosome. Three of the four rRNA molecules are produced from pre-ribosomal RNA (pre-rRNA), which is encoded by rRNA genes (rDNA) and specifically transcribed by RNA polymerase I (Pol I) in nucleoli [8–11]. The control of rRNA transcription plays a critical role in the regulation of ribosome biogenesis and cell growth [11–14]. The inhibition of rRNA transcription may offer a therapeutic strategy to block cancer cell proliferation [10, 15, 16].

KDM2A is an epigenetic protein with multiple modules [17]. KDM2A is expressed throughout the body during embryogenesis, and studies with KDM2A-KO mice suggest that KDM2A plays an essential role in embryonic development [18]. In our previous study, it was shown that KDM2A is accumulated in nucleoli and reduces rRNA transcription in serum- and glucose-free conditions [19]. We further showed that KDM2A is activated by AMP-dependent protein kinase (AMPK) sensing glucose starvation to control rRNA transcription, and that the KDM2A-dependent reduction of rRNA transcription suppresses cell proliferation [20]. These results suggest that cells have a mechanism for actively suppressing rRNA transcription via KDM2A besides passively slowing down rRNA transcription due to nutrient deficiency. However, the molecular mechanism by which KDM2A controls rRNA transcription in response to glucose starvation is unclear.

The nucleus is surrounded by the nuclear membrane, and nuclear proteins are brought to the nucleus by cellular factors through nuclear pores after production in the cytoplasm. On the other hand, specific targets of intra-nuclear structures appear to be determined by binding to the components constituting the intra-nuclear structures. The nucleolar component, to which KDM2A binds, may be involved in regulation of rRNA transcription by KDM2A. We previously reported that the CxxC-zinc finger (CxxC-zf) domain of KDM2A, which binds to unmethylated CpG sequences, is involved in KDM2A-binding to the rDNA promoter and regulation of rRNA transcription [19, 21]. These results suggest that the CxxC-zf domain may function as a nucleolar localization sequence (NoLS), because rDNA has many CpG sequences. However, KDM2A that had mutations in the CXXC-zf domain was still localized in nucleoli [20, 21], suggesting that there may be a nucleolar localization sequence in KDM2A besides the CxxC-zf domain. Until now, there have been no reports identifying a nucleolar localization sequence in KDM2A protein.

Heterochromatin protein 1 (HP1) was originally identified in the context of heterochromatin. The family of HP1 consists of highly conserved proteins. In mammals, there are three isoforms, HP1α, HP1β, and HP1γ. All HP1 isoforms bind to an H3K9me3 mark through a chromodomain (CD). HP1 also interacts with numerous proteins, many of which bind to the chromoshadow domain (CSD) of HP1 [22]. It is now clear that the biological phenomena in which HP1 is involved are not restricted to heterochromatinization but comprise many cellular activities including transcriptional activation, elongation, sister chromatid cohesion, and chromatin segregation [22]. Because loss of HP1 proteins causes chromosome segregation defects, a reduction in the levels of HP1 family members would be associated with cancer progression in humans and has been reported for many cancers [23]. However, recent reports suggested that HP1 isoforms are upregulated in some tumor tissues. For example, HP1α is highly expressed in glioma cells [24]. HP1γ is overexpressed in lung adenocarcinoma [25], non-small cell lung cancer [26], and tongue squamous cell carcinoma [27]. HP1β is highly expressed in 60% of breast cancers [28]. Therefore, the expressions and roles of HP1 family proteins in cancer cells are still not clear.

In this study, we attempted to identify the mechanism by which KDM2A was localized in nucleoli. We found that multiple regions of KDM2A protein were involved in the nucleolar localization of KDM2A. One of the regions was directly bound by HP1. We show that HP1γ was involved in the nucleolar accumulation of KDM2A, and involved in the reductions of rRNA transcription and cell proliferation on glucose starvation. It was found that HP1γ was expressed in all breast cancer tissues examined, including TNBC, as we have previously shown in KDM2A [20]. Knockdown of HP1γ in a TNBC cell line inhibited the reductions of rRNA transcription and cell proliferation on glucose starvation. These results suggest that the reduction of rRNA transcription by KDM2A requires HP1γ and may be applied to the treatment of TNBC.

## RESULTS

### KDM2A has multiple regions for nucleolar localization

**Figure 1:**
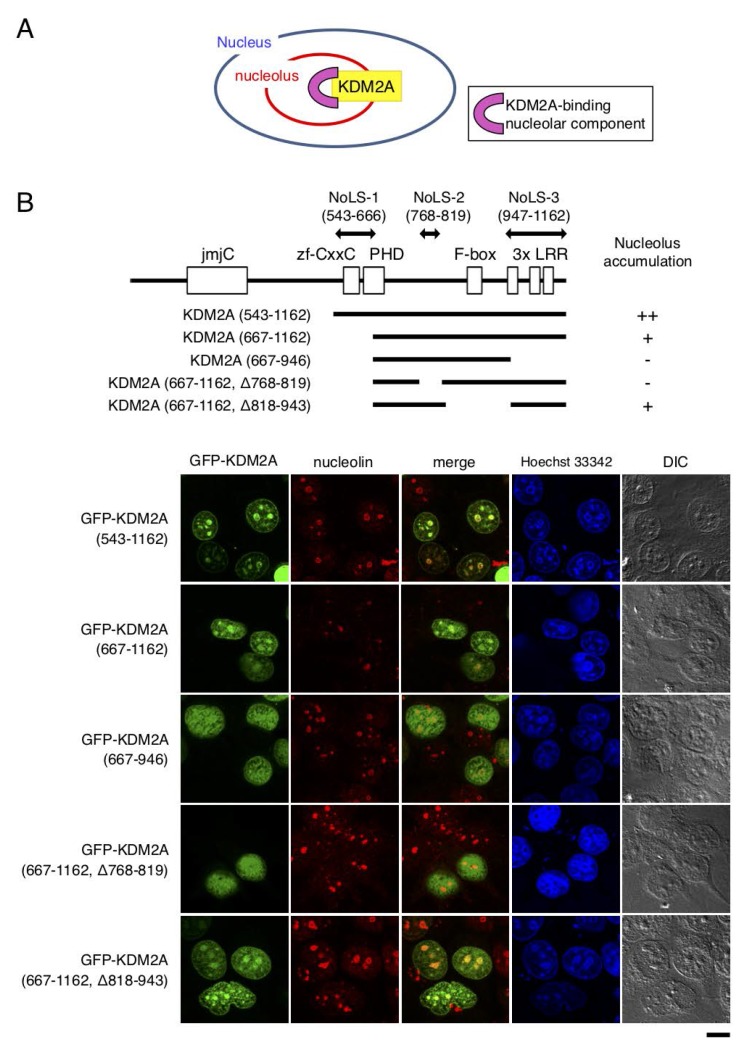
Regions responsible for nucleolar localization of KDM2A. (**A**) KDM2A as a nucleolar accumulating protein should bind to nucleolar components for its nucleolar localization. The component that binds and tethers KDM2A to the nucleolus would be involved in the regulation of KDM2A activity in the nucleolus. (**B**) Schematic diagrams of KDM2A deletion mutants, which were fused with GFP on the N-terminal side of KDM2A, are shown (upper panel). The GFP fusion proteins were expressed in MCF-7 cells, and the subcellular localizations of GFP (Green), nucleolin (Red), and nuclei (Blue) were observed (lower panel). Scale bar corresponds to 10 μm. The nucleolar localization sequences (NoLSs) of KDM2A identified in this study are shown by double-headed arrows (upper panel).

KDM2A exists in nucleoli and is involved in regulation of rRNA transcription. Identification of the region of KDM2A protein for its nucleolar localization and the component binding to the region would help understand the molecular mechanism of KDM2A-dependent control of rRNA transcription (Figure 1A). To search for the nucleolar targeting sequence in KDM2A, first we used a nucleolar localization sequence (NoLS) detection program for eukaryotic and viral proteins (http://www.compbio.dundee.ac.uk/www-nod/index.jsp) [29]. This program detected a region spanning amino acids from 561 to 582 as NoLS, which overlapped with the CxxC-zf domain (AA 563–609) (Supplementary Figure 1).

To detect the region responsible for KDM2A-nucleolar localization experimentally, a panel of deletion mutants of green fluorescence protein (GFP)-fusion KDM2A were expressed in cells. While the GFP-fusion protein with the N-terminal half of KDM2A (amino acids 1–549) was present in the cytoplasm, that of the C-terminal half of KDM2A (amino acids 543–1162), which was the same as SF-KDM2A, another protein produced by the *KDM2A* gene [19], was accumulated in nucleoli (Supplementary Figure 2), as described previously [30]. The further-deleted KDM2A (amino acids 667–1162), which had lost the region including the CxxC-zf domain and part of the PHD zinc finger, still accumulated in nucleoli, but its nucleolar accumulation efficiency was decreased (Figure 1B). Thus we designated this deleted region as nucleolar localization sequence 1 (NoLS-1 in Figure 1B). When either amino acids 768–819 or 947–1162 of KDM2A were deleted from KDM2A (amino acids 667–1162) (Figure 1B), the nucleolar accumulation was further reduced (Figure 1B). On the other hand, the deletion of amino acids 818–943 of KDM2A from KDM2A (amino acids 667–1162) did not affect the nucleolar accumulation (Figure 1B). These results suggest that amino acids 768–819 and 947–1162 of KDM2A are involved in the nucleolar accumulation of KDM2A, and we designated these regions as NoLS-2 and NoLS-3, respectively (Figure 1B). The finding that the deletion of either the NoLS-2 or NoLS-3 region from KDM2A (amino acids 667–1162) abolished the nucleolar accumulation suggests that these two regions work cooperatively to locate KDM2A (amino acids 667–1162) to the nucleolus.

### The region of nucleolar localization 2 (NoLS-2) overlaps with the region for binding of KDM2A to HP1

**Figure 2:**
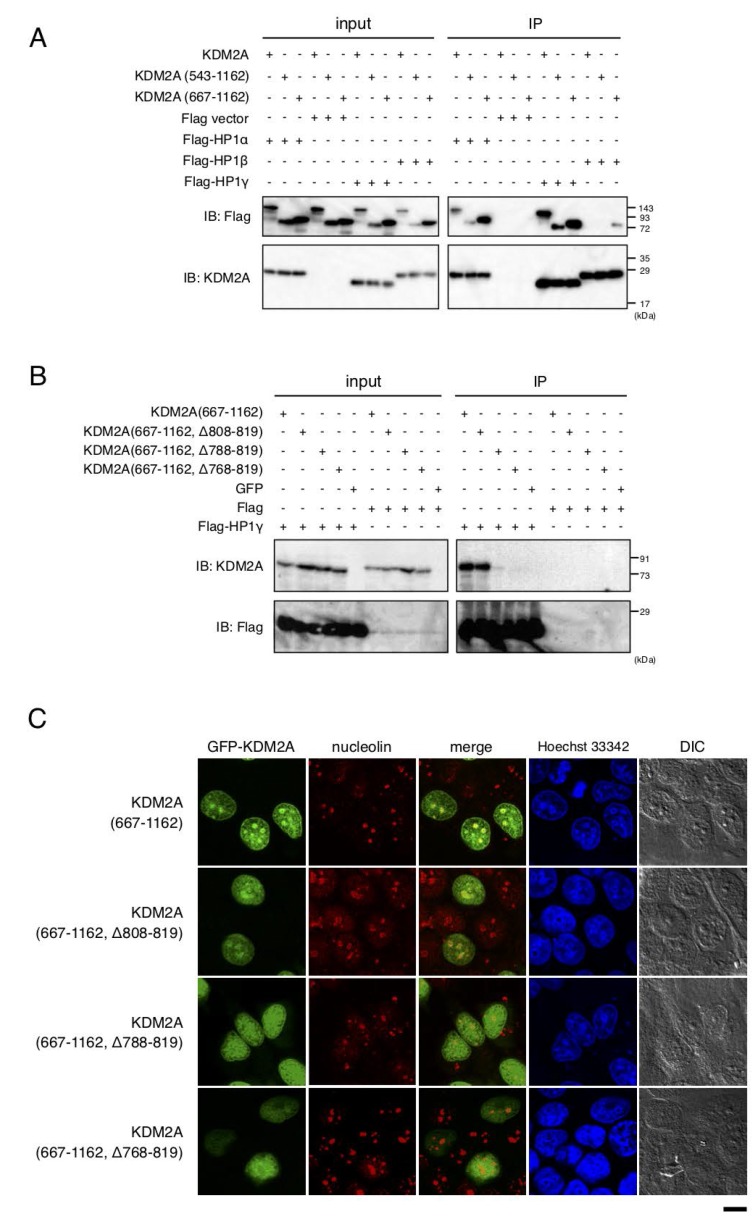
HP1-binding to KDM2A through a nucleolar localization sequence (NoLS-2). (**A**) An expression vector encoding Flag-HP1α, HP1γ, or HP1β, or the empty vector was cotransfected with an expression vector encoding KDM2A, SF KDM2A (amino acids 543-1162), or GFP fusion protein with the KDM2A fragment (amino acids 667–1162) to 293T cells. Cell lysates were immunoprecipitated with anti-Flag antibody-conjugated agarose and analyzed by Western blotting with anti-KDM2A antibody (ab99242; Abcam) and anti-Flag antibody. One tenth of input samples were also analyzed. (**B**) An expression vector encoding GFP fusion protein with KDM2A fragments, which were gradual deletions of NoLS-2, was cotransfected with an expression vector encoding Flag-HP1γ or the empty vector in 293T cells. Cell lysates were analyzed as described in A. (**C**) The GFP fusion proteins in (**B**) were expressed in MCF-7 cells and subcellular localizations of GFP (Green), nucleolin (Red), and nuclei (Blue) were observed. Scale bar corresponds to 10 μm.

It was reported that three mammalian HP1 isoforms interact with KDM2A [31–33]. To check the interaction between KDM2A and HP1 isoforms, Flag-tagged HP1α, HP1β, or HP1γ was co-expressed with KDM2A in cells, and immunoprecipitated using an anti-Flag antibody. While KDM2A was co-precipitated with HP1α, HP1β, or HP1γ, the efficiencies of the co-precipitation with the three HP1 isoforms were different (Figure 2A). Among the three isoforms, HP1γ had the highest efficiency of co-precipitating KDM2A and SF-KDM2A (amino acids 543–1162), HP1α was moderate, and HP1β was the least efficient. GFP-KDM2A (amino acids 667–1162) was co-precipitated with either HP1γ or HP1α with similar efficiencies, and with less efficiency with HP1β. Our results were basically consistent with previous reports [31, 32], but suggested that the three isoforms had different efficiencies in binding to KDM2A in our experimental conditions.

Next, whether the NoLSs identified here were required for binding of HP1γ to KDM2A was tested. While the deletion mutants of KDM2A (667–1162) that lack NoLS-3 and amino acids 818–943 regions were co-precipitated with Flag-HP1γ (Supplementary Figure 3), the graded deletions of the NoLS-2 region (amino acids 768–819) from GFP-KDM2A (amino acids 667–1162) gradually decreased the efficiency of co-precipitation with Flag-HP1γ (Figure 2B). Graded deletions of the NoLS-2 region (amino acids 768–819) from GFP-KDM2A (amino acids 667–1162) also gradually decreased the efficiency of nucleolar accumulation of the fragment (Figure 2C, summarized in Supplementary Figure 4). The results suggest that the interaction of KDM2A with HP1γ contributes to the localization of KDM2A to the nucleoli.

When HP1γ was immunostained in cells expressing GFP-KDM2A (amino acids 667–1162), the signals for HP1γ were observed throughout the nuclei with strong dots (Supplementary Figure 5). The signals for GFP-KDM2A (amino acids 667–1162) were partly overlapped with and surrounded by the signal for HP1γ. These data do not conflict with the hypothesis that HP1γ contributes to nucleolar localization of KDM2A, but is not the sole factor determining the nucleolar localization of KDM2A (amino acids 667–1162).

### HP1γ directly binds to KDM2A using valine 801 in LxVxL motif of KDM2A as a critical amino acid

**Figure 3:**
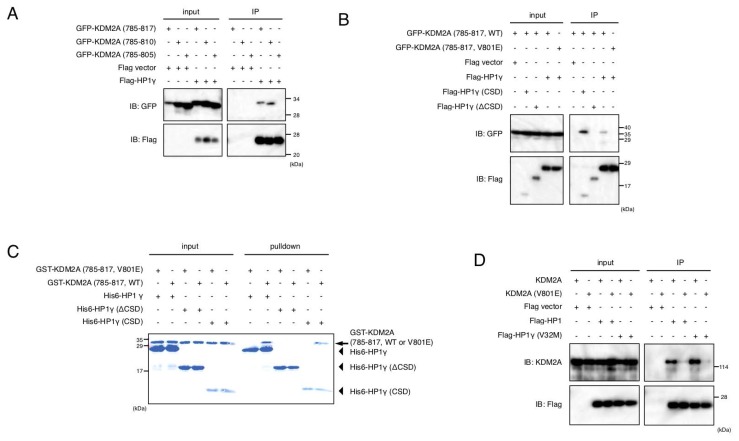
Valine 801 of KDM2A has a critical role in binding of KDM2A to HP1. (**A**) An expression vectors encoding GFP fusion proteins fused with KDM2A (amino acids 785–817), KDM2A (amino acids 785–810), or KDM2A (amino acids 785–805) were cotransfected with an expression vector encoding Flag-HP1γ or the empty vector in 293T cells. Cell lysates were immunoprecipitated by anti-Flag antibody, and analyzed by Western blotting usng anti-GFP and anti-Flag antibodies. One tenth of input samples were also analyzed. (**B**) An expression vector encoding GFP fusion protein with KDM2A (amino acids 785–817) or the KDM2A fragment (amino acids 785–817, V801E) was cotransfected with an expression vector encoding Flag-HP1γ, Flag-HP1γ (CSD) or Flag-HP1γ (ΔCSD), or the empty vector in 293T cells. Cell lysates were analyzed as in (**A**). (**C**) Recombinant His-tagged HP1γ was incubated with the recombinant KDM2A fragment (amino acids 785–817) fused with GST or the recombinant KDM2A fragment (amino acids 785–817, V801E) fused with GST, followed by the His-tag protein collecting system, TALON. Collected proteins were analyzed by SDS-PAGE. The arrow indicates the position of GST-KDM2A fragments. The arrowheads indicate the positions of His-HP1γ or His-HP1γ mutants. (**D**) An expression vector encoding KDM2A or mutant KDM2A (V801E) was cotransfected with an expression vector encoding Flag-HP1γ or Flag-HP1γ (V32M) or the empty vector in 293T cells. Cell lysates were analyzed as in Figure 2A.

Next, the specific site of KDM2A required for binding HP1γ was determined. KDM2A (amino acids 742–817) bound to HP1γ (Supplementary Figure 6). A series of further deletions of the KDM2A fragment were tested for binding to HP1γ, and revealed that KDM2A (amino acids 785–810) was sufficient for effective binding to HP1γ (Figure 3A and Supplementary Figures 7 and 8). There are two domains, the chromodomain (CD) and the chromoshadow domain (CSD), in HP1 protein. While CD of HP1 binds to a histone mark H3K9me3, CSD binds the majority of HP1-interacting proteins recognizing the PxVx(M/L/V) motif [34–36]. It was found that HP1γ lacking CSD (Flag-HP1γ ΔCSD) did not bind to KDM2A (amino acids 785–817), while HP1γ lacking CD (Flag-HP1γ CSD) still bound to the KDM2A fragment (Figure 3B). Although there are no PxVx(M/L/V) motifs in KDM2A (amino acids 785–810), there is an LxVxL motif, which was rarely reported to bind HP1 protein and conserved in KDM2A of vertebrates (Supplementary Figure 9) [34, 37–39]. Substitution of the central valine with glutamic acid in the LxVxL motif eliminated the interaction (Figure 3B), suggesting the hydrophobic amino acid, valine, is required for the interaction of KDM2A with HP1γ. Next, whether the mutation of valine 801 of KDM2A affected the subcellular localization of the KDM2A fragment was examined. While GFP-KDM2A (amino acids 667–1162) was accumulated in nucleoli, GFP-KDM2A (amino acids 667–1167, V801E) was not (Supplementary Figure 10). These results show that the central valine in the LxVxL motif requires nucleolar accumulation of the KDM2A fragment.

To determine if HP1γ directly binds to the KDM2A fragment, we carried out an *in vitro* binding assay by mixing recombinant His-tagged HP1γ and recombinant GST fusion KDM2A (amino acids 785–817), followed by an His-tag collecting system. It was found that HP1γ and CSD of HP1γ bound to the KDM2A fragment (amino acids 785–817) but did not bind to the mutant KDM2A fragment (amino acids 785–817, V801E) (Figure 3C). Together, these results indicate that HP1γ directly binds to KDM2A using valine 801 in the LxVxL motif of KDM2A as a critical amino acid.

When full-length KDM2A or KDM2A with the mutation at valine 801 to glutamic acid (mutant KDM2A (V801E)) was co-expressed with Flag-HP1γ in cells, KDM2A but not the mutant KDM2A (V801E) was co-purified with Flag-HP1γ (Figure 3D). KDM2A was also co-purified with Flag-HP1γ (V32M) (Figure 3D), which had a point mutation at valine 32 and lost the binding activity to H3K9me3, confirming that CD was not involved in the binding of KDM2A to HP1γ. These results show that valine 801 in the LxVxL motif is a critical amino acid for binding of full-length KDM2A to HP1γ.

### HP1γ is involved in accumulation in nucleolus

**Figure 4:**
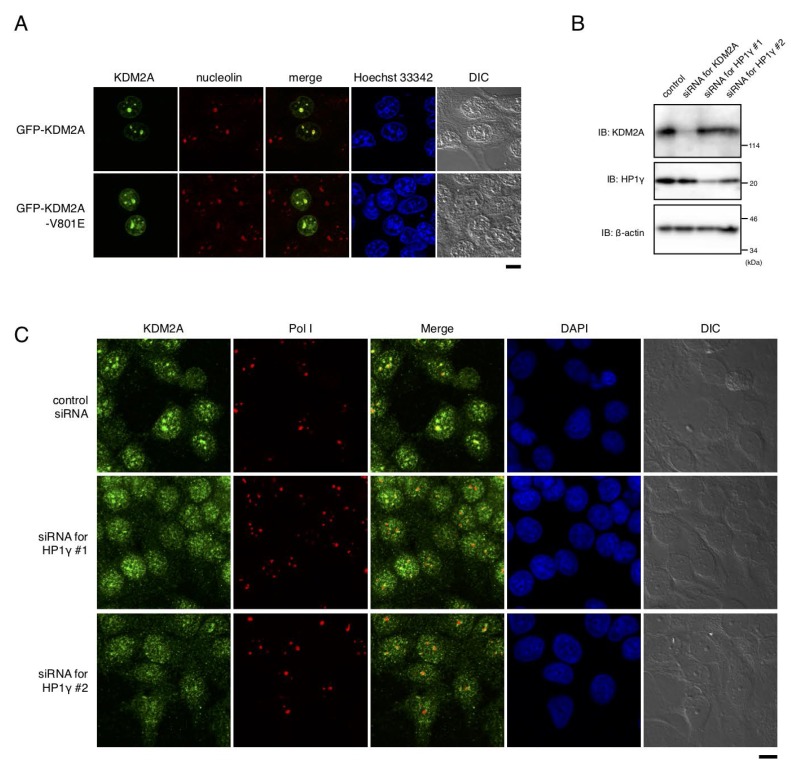
HP1γ is involved in nucleolar accumulation and binding to rDNA promoter of KDM2A. (**A**) MCF-7 cells were transfected with an expression vector encoding GFP (Green) fusion KDM2A or KDM2A (V801E), and stained with anti-nucleolin antibodies (Red) and Hoechet 33342 (Blue). The signals were observed through a fluorescent microscope. Most of the signals for KDM2A and mutant KDM2A (V801E) were colocalized with those for nucleolin, but the efficiency of nucleolar accumulation of KDM2A (V801E) was lower than that for KDM2A. Scale bar corresponds to 10 μm. (**B**) MCF-7 cells were transfected with siRNAs for KDM2A or for HP1γ (HP1γ#1 and HP1γ#2), and cell lysates were analyzed by Western blotting. (**C**) Cells treated as in (**B**) were stained with anti-KDM2A antibody (Green), anti-RNA polymerase I antibody (Red), and DAPI (Blue) and observed by fluorescent microscopy and differential interference contrast microscopy. Scale bar corresponds to 10 μm.

When the mutant KDM2A (V801E) fused with GFP was expressed, the protein signals were still detected in nucleoli, but the nucleolar accumulation efficiency was decreased compared to that of wild-type KDM2A (Figure 4A). Further, a knockdown of HP1γ by siRNAs for HP1γ (Figure 4B) in MCF-7 cells reduced nucleolar accumulation of endogenous KDM2A (Figure 4C). The expression level of KDM2A was not affected by a knockdown of HP1γ (Figure 4B). A knockdown of KDM2A did not affect the nucleolar accumulation of RNA polymerase I or the morphology of nucleoli detected by differential interference contrast microscopy (Figure 4C). These results indicate that HP1γ is specifically involved in the nucleolar localization of KDM2A.

### HP1γ is involved in KDM2A to reduce rRNA transcription and cell proliferation on glucose starvation

**Figure 5:**
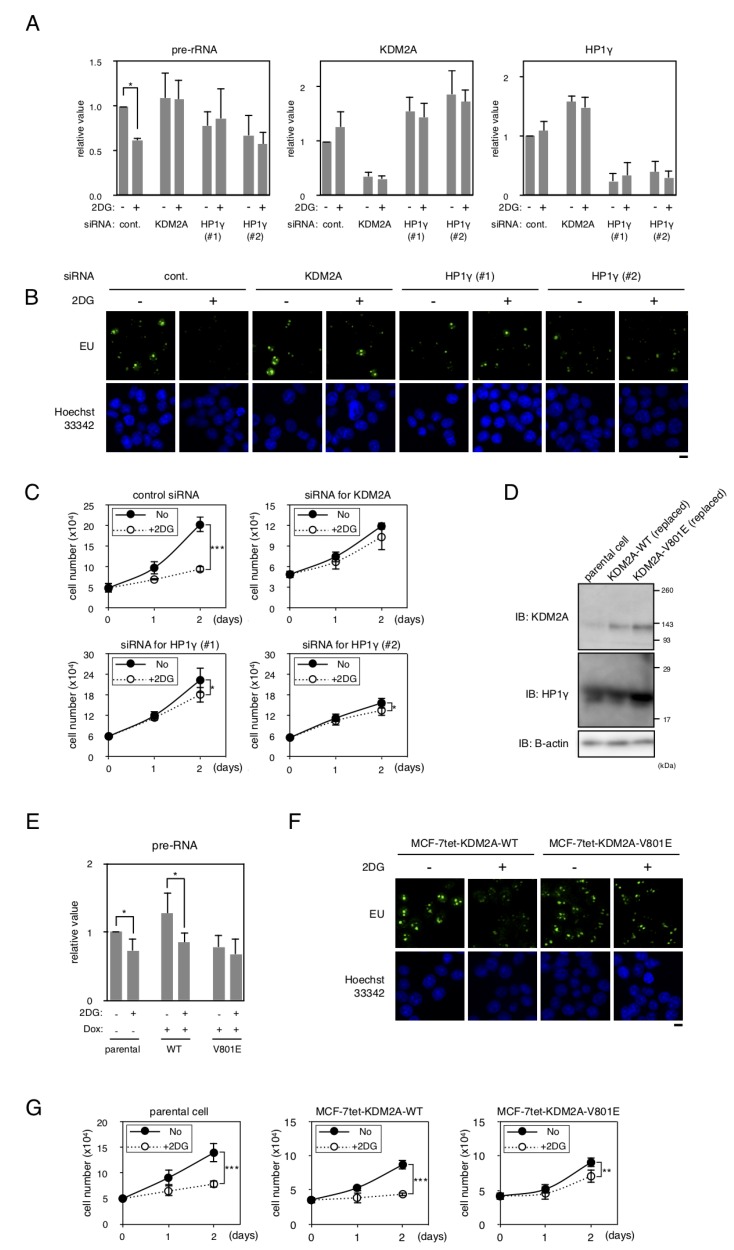
HP1γ is involved in KDM2A-dependent reductions of rRNA transcription and cell proliferation on glucose starvation. (**A**) MCF-7 cells transfected with control siRNA (control), HP1γ siRNA (HP1γ#1 and HP1γ#2), or KDM2A siRNA (KDM2A) for three days were replated in the growth medium. Next day, cells were cultured in the presence or absence of 2 mM 2DG for two hours, and total RNA was isolated and the pre-rRNA, KDM2A mRNA, and HP1γ mRNA were detected by RT-PCR. The results were normalized by the values of Polr2a mRNA. (**B**) The effects of a knockdown of HP1γ or KDM2A on rRNA transcription were investigated by metabolic labelling assay. Cells transfected as in (**A**) were replated on coverlips, and metabolically labeled with 5-ethynyl uridine (EU), as described in materials and methods. (**C**) Cells transfected as in (**A**) were replated. Next day, cells were cultured in the presence or absence of 2 mM 2DG for two days. At the indicated days, cell numbers in three independent wells were counted. Mean values with the standard deviations are indicated. (**D**) MCF-7 cell lines in which wild-type KDM2A or the mutant KDM2A (V801E) can be induced under the tet-on system (MCF-7tet-KDM2A and MCF-7tet-V801E, respectively) were established. The cells were transfected with siRNA for KDM2A and treated with doxycycline (Dox) for two days to overexpress the exogenous KDM2As. Cell lysates were analyzed by Western blotting using anti-KDM2A antibody or anti-HP1γ antibody. β-actin was also detected as a loading control. Parental cells transfected with control siRNA were used as a control (parental cells). (**E**) After treatment described in (**D**), cells were replated and cultured for two days. Cells were further cultured for two hours in the presence or absence of 2 mM 2DG. Total RNA was isolated and the pre-rRNA was measured by RT-PCR. Polr2a mRNA was also detected to normalize the results. (**F**) The effect of the V801E mutation of KDM2A on rRNA transcription were investigated by metabolic labelling assay. Cells that were replaced endogenous KDM2A with exogenous wild type KDM2A or the mutant KDM2A (V801A) were replated on coverslips, and metabolically labeled with EU, as described in materials and methods. (**G**) After treatment described in (**D**), cells were replated and cultured for two days. Cells were further cultured for two days in the presence or absence of 2 mM 2DG, and cell numbers were counted at days indicated. The results from three independent wells were averaged. Mean values with the standard deviations are indicated. *, P <0.05; **, P <0.005; ***, P<0.0001.

We investigated whether HP1γ is involved in controlling the activities of KDM2A to regulate rRNA transcription in MCF-7 cells. When cells were treated with 2 mM 2-deoxyglucose (2DG), the amount of pre-rRNA was reduced. A knockdown of either HP1γ or KDM2A suppressed the reduction of pre-rRNA (Figure 5A), suggesting that HP1γ and KDM2A are involved in the reduction of rRNA transcription. These results were validated by metabolic labelling assay. First cells were metabolically labeled by 5-ethynyl uridine (EU), and then stained by anti-RNA polymerase I antibody. The signals of EU were overlapped with the signals for RNA polymerase I (Supplementary Figure 11), showing that the signals of EU staining represented rRNA transcription in the conditions. While the 2DG treatment reduced the signals of EU, a knockdown of HP1γ or KDM2A alleviated the reduction of the EU signals by 2DG (Figure 5B). These results confirm that HP1γ and KDM2A contribute to the reduction of rRNA transcription induced by 2DG.

We previously suggested that the KDM2A-dependent reduction of rRNA transcription reduced proliferation of cells [20]. When MCF-7 cells were cultured in the presence of 2 mM 2DG for one and two days, cell numbers were reduced to about 70% and 46%, respectively (Figure 5C). Treatment of cells with siRNA for KDM2A or HP1γ (HP1γ#1 or HP1γ#2) suppressed the reduction of cell number after one day, and maintained cell numbers at more than 80% after two days compared to those without the treatment of 2DG (Figure 5C). These results suggest that HP1γ is required for the reductions of rRNA transcription and cell proliferation on glucose starvation.

To test whether the mutation at valine 801 of KDM2A affected the activities of KDM2A to control rRNA transcription, the wild-type KDM2A and the mutant KDM2A (V801E) were overexpressed after knockdown of endogenous KDM2A. As shown in Figure 5D, the mutant KDM2A (V801E) was overexpressed at a level comparable to that of the wild-type KDM2A in cells. In cells overexpressing the wild-type KDM2A, the 2DG treatment reduced the amount of pre-rRNA similarly to that observed in parental cells (Figure 5E). On the contrary, in cells overexpressing the mutant KDM2A (V801E), reduction of pre-rRNA by 2DG was hardly observed (Figure 5E). The results were confirmed by metabolic labelling assay using EU (Figure 5F). These results suggest that valine 801 is required for the activity of KDM2A to suppress the rRNA transcription in response to glucose starvation.

Next, we investigated whether valine 801 of KDM2A was involved in the control of cell proliferation under glucose starvation conditions. When parental cells and cells overexpressing wild KDM2A were cultured in the presence of 2 mM 2DG for two days, cell numbers were reduced to 55% (Figure 5G). On the other hand, when the cells overexpressing mutant KDM2A (V801E) were cultured in the presence of 2 mM 2DG, cell numbers were reduced only to 75% in two days (Figure 5G). These results suggest that the change of rRNA transcription in glucose starvation conditions was reflected in the change of cell proliferation in these experimental conditions.

### HP1γ is continuously expressed in breast cancers

**Figure 6:**
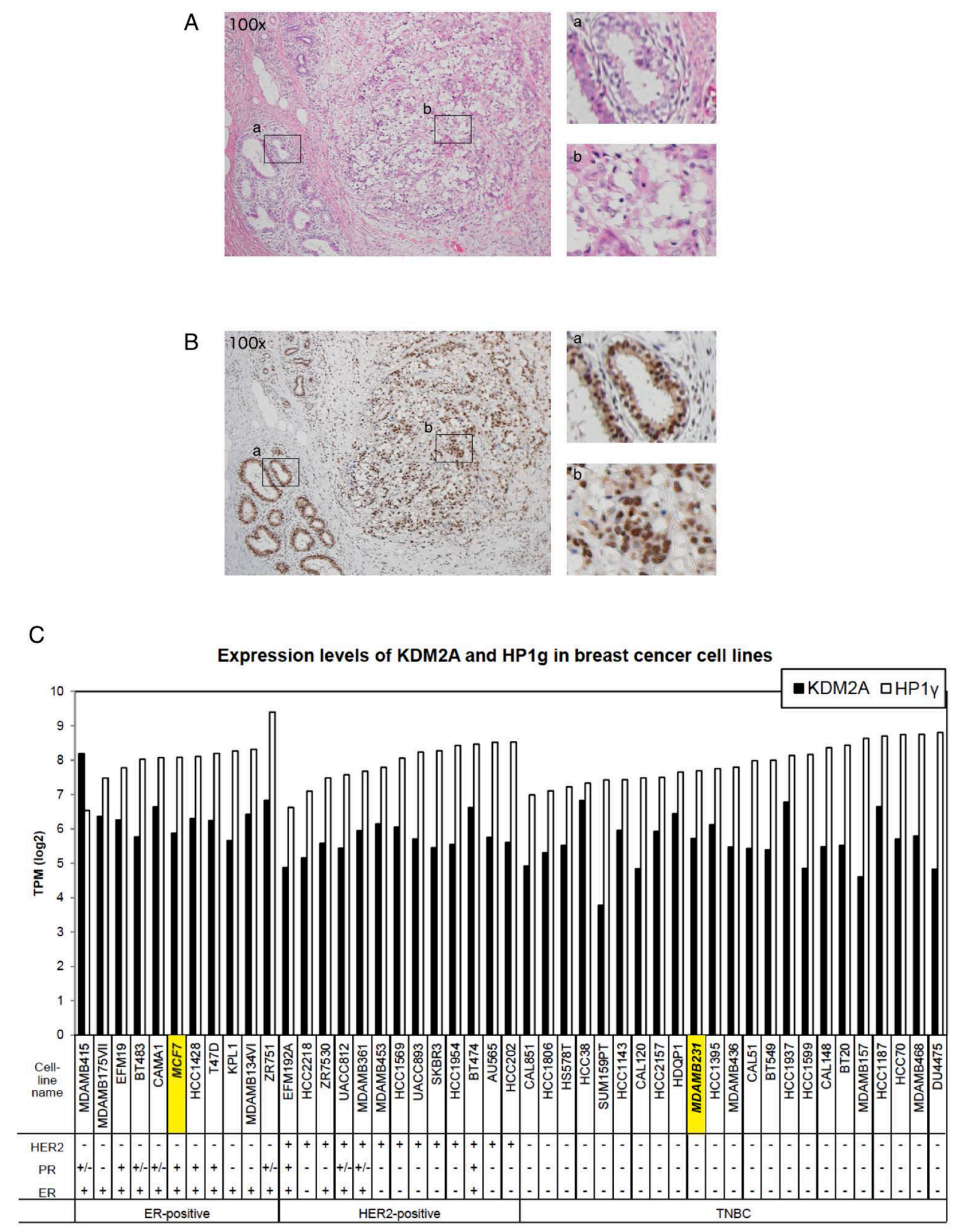
HP1γ expression is maintained in breast cancer tissues. (**A**) Breast cancer tissue with scirrhous carcinoma (hematoxylin & eosin staining, x100). (**B**) A serial section of A stained by anti- HP1γ antibody, showing the expression of HP1γ in both neoplastic and non-neoplastic areas (x100). Positive staining is brown and counterstained nuclei are blue. Enlargements are also shown (right upper, non-neoplastic area; right lower, carcinoma area). (**C**) Expression analysis of HP1γ and KDM2A mRNAs in breast cancer cell lines using publically available databases. The expression data (Expression public 19Q1) were downloaded from Cancer Dependency Map project (Depmap) site in Broad institute, and expression of HP1γ and KDM2A mRNAs in breast cancer cell lines were analyzed.

The expression of HP1 family members had been reported to be reduced in many cancers, and would be associated with cancer progression in humans, because HP1 is involved in chromatin segregation [23]. However, recent reports suggested that HP1 isoforms were upregulated in some tumor tissues. In breast cancers, it was reported that about 60% of samples examined showed high HP1β expression and about 40% of them showed no or low expression of each HP1 subtype [28]. Therefore, the expressions of HP1γ in cancer cells are controversial. To investigate the expression of HP1γ in breast cancer tissues, tumors from surgical breast cancer specimens including papillotubular, solid-tubular, scirrhous, mucinous, and micropapillary carcinomas were resected, and HP1γ was detected immunohistochemically. Hematoxylin and eosin staining was used to demarcate tumor areas. The section shown in Figure 6A is breast cancer tissue with scirrhous carcinoma and a non-neoplastic area. Figure 6B shows marked staining of HP1γ in both neoplastic and non-neoplastic areas. Staining of HP1γ was found in the nuclear regions of cells in both neoplastic and non-neoplastic areas. Staining indexes of HP1γ in 39 neoplastic areas were determined (Table 1). All specimens scored 6–8 on a scale of 8 on HP1γ staining (HP1γ score), and no specimen with HP1γ scored below 6. These results demonstrate that the expression of HP1γ in breast carcinomas was maintained during carcinogenesis, as well as that of KDM2A [20].

**Table 1:** Expression of HP1γ protein in breast cancer tissues

	Papillotubular		Solid-tubular	Scirrhous			Mucinous		Micropapillary
HP1γ score	7	8	8	6	7	8	7	8	8
Number	2	16	3	1	2	11	1	1	2
Strong expression of peripheral zone	2	0	0	1	1	0	1	0	0
HER2 0	0	8	0	1	2	3	1	0	0
1	1	3	1	0	0	1	0	1	0
2	0	2	2	0	0	3	0	0	1
3	1	3	0	0	0	4	0	0	1
ER −	0	4	0	0	0	2	0	0	1
+	2	12	3	1	2	9	1	1	1
PgR −	1	5	1	1	0	3	1	0	1
+	1	11	2	0	2	8	0	1	1
Triple Negative	0	2	0	0	0	0	0	0	0

We further analyzed the levels of HP1γ and KDM2A mRNAs in breast cancer cell lines, using publically available databases. The results show that most of breast cancer cell lines express HP1γ and KDM2A mRNAs similarly to those of MCF-7 cells, and triple negative breast cancer cell lines, including MDA-MB-231, also express HP1γ and KDM2A (Figure 6C). These results are consistent with our observation that the expression of both HP1γ and KDM2A are maintained in breast cancers (Figure 6B).

### HP1γ is involved in reduction of rRNA transcription in triple-negative breast cancer cell line MDA-MB-231

**Figure 7:**
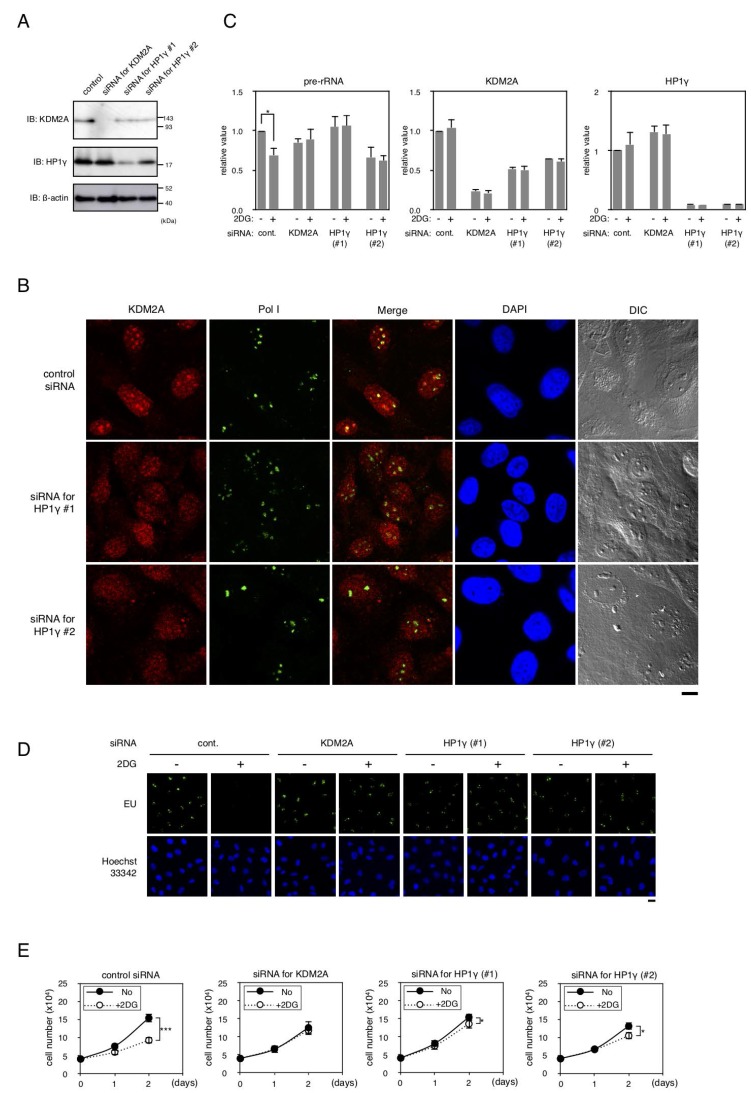
Effects of HP1γ on KDM2A activities in MDA-MB-231 cells. (**A**) MDA-MB-231 cells were transfected for two days with siRNAs for KDM2A or HP1γ (HP1γ#1 and HP1γ#2), and cell lysates were analyzed by Western blotting. (**B**) Cells transfected as in (**A**) were stained with anti-KDM2A antibody (Red), anti-RNA polymerase I antibody (Green), and DAPI (Blue) and observed by fluorescent microscopy and differential interference contrast microscopy. Scale bar corresponds to 10 μm. (**C**) Cells transfected as in (**A**) were replated in the growth medium. Next day, cells were cultured in the presence or absence of 3 mM 2DG for two hours. Total RNA was isolated and the pre-rRNA, KDM2A mRNA, and HP1γ mRNA were detected by RT-PCR. The results were normalized to the values of Polr2a mRNA. (**D**) The effects of a knockdown of HP1γ or KDM2A on rRNA transcription were investigated by metabolic labelling assay. Cells transfected as in (**A**) were replated on coverslips, and metabolically labeled with EU, as described in materials and methods. (**E**) Cells transfected as in (**A**) were replated and cultured in the presence or absence of 3 mM 2DG for two days. On the indicated days, cell numbers in three independent wells were counted. Mean values with the standard deviations are indicated. *, P <0.05; ***, P<0.0001.

The expression of HP1γ was reduced by the specific siRNAs in a triple negative breast cancer cell line MDA-MB-231 (Figure 7A). A knockdown of HP1γ partly reduced the amount of KDM2A protein in cells (Figure 7A). As shown in Figure 7B, a knockdown of HP1γ reduced nucleolar accumulation of endogenous KDM2A, but hardly affected the nucleolar accumulation of RNA polymerase I or morphology of nucleoli. To fairly compare the signals in cells with an HP1γ knockdown to those in control cells, the KDM2A signals in HP1γ knockdown cells were enhanced (Figure 7B). These results indicate that HP1γ is involved in the nucleolar localization of KDM2A in MDA-MB-231 cells. In MDA MB-231 cells, an HP1γ knockdown reduced the nucleolar accumlation of KDM2A (Figure 7B), as well as the expression of KDM2A protein and KDM2A mRNA (Figure 7A and 7C). These results suggest that HP1γ increases the nucleolar concentration of KDM2A by two ways in MDA-MB-231 cells, elevations of the nucleolar accumulation and the expression of KDM2A, both of which would contribute to a KDM2A-dependent reduction of rRNA transcription.

Next we investigated whether HP1γ is involved in controlling rRNA transcription on glucose starvation. When cells were treated with 3 mM 2DG, the amount of pre-rRNA was reduced, and a knockdown of either HP1γ or KDM2A suppressed the reduction of pre-rRNA induced by the 2DG treatment (Figure 7C). The results were confirmed by metabolic labelling assay using EU (Figure 7D).

When MDA-MB-231 cells were cultured in the presence of 3 mM 2DG for one and two days, cell numbers were reduced to about 70% and 46%, respectively (Figure 7E). Treatment of cells with siRNA for KDM2A or HP1γ (HP1γ#1 or HP1γ#2) suppressed the reduction of cell number after one day and maintained cell numbers at more than 80% after two days compared to those without the 2DG treatment (Figure 7E). Together, these results suggest that HP1γ is required for the reductions of rRNA transcription and cell proliferation on glucose starvation in MDA-MB-231 cells.

## DISCUSSION

To investigate the molecular mechanisms of KDM2A function in the nucleolus, we dissected the amino acid sequence of KDM2A to identify regions responsible for its nucleolar localization (Figure 1). We found that three regions were involved in the nucleolar localization of KDM2A (Figure 2), and one of the regions was directly bound by HP1γ (Figure 3). A knockdown of HP1γ or replacement of endogenous KDM2A with a mutant KDM2A that did not bind HP1γ reduced the nucleolar accumulation of KDM2A (Figure 4), and suppressed the reduction of the rRNA transcription on glucose starvation (Figure 5). Because rRNA transcription occurs in nucleoli, it is plausible that the elevated nucleolar accumulation of KDM2A by HP1γ supports the KDM2A-dependent regulation of rRNA transcription in nucleoli. From these results, we concluded that HP1γ is involved in the KDM2A-mediated reduction of rRNA transcription on glucose starvation. A knockdown of HP1γ or replacement with the mutant KDM2A also suppressed the reduction of cell proliferation on glucose starvation (Figure 5). Because cell proliferation is correlated with rRNA transcription [20], these results are consistent with our conclusion. Together, our results uncovered a novel function of HP1γ, the control of rRNA transcription on glucose starvation.

Among women, breast cancer is the most commonly diagnosed cancer and the leading cause of cancer death worldwide [1, 2]. Although the expression of HP1 family members had been previously reported to be reduced in many cancers [23, 28], we observed here that HP1γ was expressed in all breast cancer tissues examined (Figure 6 and Table 1) and all breast cancer cell lines listed in data bases (Figure 6). We previously demonstrated that KDM2A is expressed in breast cancer tissues [20]. Because the potential therapeutic effects of specific inhibitors of rRNA transcription without the effects of the health and body weights of mice were reported [40–43], our results suggest that the regulation of the KDM2A- and HP1γ-dependent mechanisms controlling rRNA transcription may be applicable to the treatment of breast cancer. Among breast cancers, patients with TNBC have a poor prognosis compared to the other subtypes of breast cancer [3, 6, 7]. While several novel therapeutic possibilities for TNBC were recently proposed [3–5], the heterogeneous nature of TNBC is standing in the way of therapy, and additional treatments were expected to be developed for TNBC. We showed that KDM2A [20] and HP1γ (Figure 6 and Table 1) are expressed in breast cancer tissues, including TNBCs, and that KDM2A with HP1γ reduced rRNA transcription and cell proliferation on glucose starvation in a TNBC line MDA-MB-231 (Figure 7). Analysis using publically available databases show that HP1γ and KDM2A are expressed in multiple TNBC cell lines with comparable levels to those of MDA-MB-231 cells (Figure 6).

We previously demonstrated that AMPK, which is activated by glucose starvation, activates KDM2A to reduce rRNA transcription [20]. Recently, AMPK activation was proposed to be applied for the treatment of cancers [44]. AMPK activators include the pharmacologic activators such as 5-aminoimidazole-4-carboxamide ribonucleotide (AICAR) and metformin, and also other chemicals. Some of the compounds may effectively suppress cell proliferation of TNBC cells through KDM2A activation and be appliciable to the treatment of TNBC.

We found that at least three regions, NoLS-1, -2, and -3, were involved in the nucleolar localization of KDM2A (Figure 1). NoLS-1 includes a CxxC-zf domain, through which KDM2A binds to an rDNA promoter having unmethylated CpG dinucleotide sequences (14). NoLS-2 bound to HP1γ (Figure 2). NoLS-3 contains leucine-rich repeats (LRR), which were suggested to have a protein-binding activity. These factors cooperatively function to localize KDM2A to the nucleolar region and may regulate the function of KDM2A. Further identification of the factors targeting KDM2A to the nucleolus would help to understand the mechanism of rRNA transcription regulation by KDM2A, which may develop effective treatments for TNBC.

## MATERIALS AND METHODS

### Cells and cell culture

Cells were cultured at 37° C in an atmosphere containing 5% CO_2_ and 100% humidity. The human breast adenocarcinoma cell line MCF-7 was cultured in RPMI1640 medium (Nacalai Tesque, Kyoto, Japan) supplemented with 10% fetal calf serum (FCS). MCF-7 cells having the tet-on transcription factor [21] were cultured in Dulbecco’s modified Eagle’s medium (DMEM, Cat# D5796, Sigma-Aldrich Co., St. Louis, MO, USA) supplemented with 10% FCS. Mammalian expression plasmids were introduced into cells using FuGENE6 transfection reagent (Promega, Madison, WI, USA) according to the manufacturer’s instructions. MCF-7tet-on cells (parent), which were G418 resistant, were transfected with ptet-KDM2A, a ptet-KDM2A(V801E) mutant, plus pActHyg, which confers hygromycin resistance, and cultured in the presence of 150 to 250 μg/ml hygromycin and 200 μg/ml G418. Selected colonies were picked up and cultured for 24 h in the presence of 1 μg/ml doxycycline (Dox), and the expression of KDM2A or its mutant protein was detected by indirect immunofluorescence and Western blotting using an anti-KDM2A antibody. To replace endogenous KDM2A with ectopically overexpressed KDM2As, cells were treated with KDM2A siRNA [19] and then cultured in the presence of 0.5 μg/ml Dox. The human breast adenocarcinoma cell line MDA-MB-231 was cultured in 10FCS-DMEM.

### Plasmids

KDM2A expression vectors were described previously [21]. The cDNA encoding mutant KDM2A(V801E) was constructed using a KOD-Plus Mutagenesis Kit, a PCR-based mutation introducing system (Toyobo, Osaka, Japan), according to the manufacturer’s instructions and subcloned into a pCAGGS mammalian expression vector. The plasmids expressing GFP fusion KDM2A mutants were constructed in a pEGFP expression vector (TaKaRa Bio Inc., Ohtsu, Japan), by PCR amplification with appropriate primers. Plasmids expressing KDM2A fragments fused with GST were constructed in pGEX-3X (Amersham Biosciences, Buckinghamshire, UK). The cDNAs for human HP1α were described previously [36, 45]. The cDNAs for human HP1β (GeneBank Accession No. AB587426) and human HP1γ (GeneBank Accession No. AB463500) were obtained from Kazusa DNA Research Institute (Kisarazu, Japan). The cDNA for the HP1γ used encodes 183 amino acids. The cDNA for HP1γ (ΔCSD) and HP1γ (CSD) encoding amino acids 1–115 and amino acids 110–183, respectively, were constructed by PCR amplification with appropriate primers. The cDNA encoding HP1γ with valine 32 replaced by methionine, HP1γ (V32M), was constructed using a KOD-Plus Mutagenesis Kit (Toyobo) according to the manufacturer’s instructions. HP1γ (V32M) lost binding activity to H3K9me3. The cDNAs for HP1s were subcloned into a pCAGGS mammalian expression vector that had a flag sequence added to the N-terminus of HP1s. The plasmids expressing His-tag HP1γ and its mutants were constructed in a pCold I vector (TaKaRa Bio). The nucleotide sequences in all plasmids here were confirmed by DNA sequencing.

### Antibodies

Mouse monoclonal anti-nucleolin antibody (Santa Cruz Biotechnology, Santa Cruz, CA, USA, C23 (MS-3), sc-8031), mouse monoclonal anti-β-actin antibody (Sigma, AC-15), mouse monoclonal anti-Flag antibody (ANTI-FLAG M2 Monoclonal Antibody, Sigma, F1804), mouse monoclonal anti-HP1γ antibody (Sigma, MAB3450), mouse monoclonal anti-RNA polymerase I antibody (Santa Cruz Biotechnology, anti-RPA194 antibody (C-1), sc-48385), rabbit polyclonal anti-GFP antibody (Santa Cruz, sc-8334), goat anti-rabbit IgG-HRP (Santa Cruz, sc-2054), goat anti-mouse IgG-HRP (Santa Cruz #sc-2005), Alexa 568-conjugated goat anti-mouse IgG (H+L) (Thermo Fisher Scientific, Waltham, MA, USA, A11004), Alexa 488-conjugated goat anti-mouse IgG (H+L) (Thermo Fisher, A11029), and Alexa 568-conjugated goat anti-rabbit IgG (H+L) (Thermo Fisher, A11036), mouse monoclonal anti-dimethylated histone H3 lys36 antibody (Active Motif, California, USA*,* MABI0332), and rabbit polyclonal anti-histone H3 antibody (Abcam, Cambridge, UK, ab1791), were purchased. The anti-KDM2A antibody was described previously [19]. The anti-Fbxl11 (KDM2A) antibody (Abcam, ab99242) was purchased and used to detect the C-terminal half of KDM2A.

### Immunofluorescence staining and Western blotting

For indirect immunofluorescence staining, cells grown on glass coverslips were fixed in methanol for 30 min at –20° C and incubated in 1% skim milk in PBS at 37° C. The first antibodies were added and incubated for 60 min at 37° C. After cells were washed three times in 0.1% skim milk in PBS, the Alexa-conjugated second antibody (Alexa 488-conjugated anti-mouse IgG, and/or Alexa 568-conjugated anti-rabbit IgG), containing 0.4 μg/ml 4›,6-diamidino-2-phenylindole (DAPI) or 2 μg/ml Hoechst 33342 was added, incubated for 60 min at 37° C, and washed three times with 0.1% skim milk in PBS. Finally, cells were embedded in Immunon (Thermo Fisher) and observed via confocal fluorescence microscopy and differential interference contrast microscopy.

For Western blotting, cells were trypsinized and extracted in 3% SDS solution containing 100 mM Tris-HCl, pH 6.8, 0.1 M DTT, and 20% glycerol. Cell extracts were separated on SDS-PAGE and transferred to a microporous PVDF membrane (Merck Millipore, Darmstadt, Germany). After treatment with antibodies, bands were detected using an Immobilon Western System (Merck Millipore, WBKLS0100).

### Immunoprecipitation

Cells plated on dishes were collected using PBS containing 2.5 mg/ml trypsin and 1 mM EDTA solution, and suspended in 0.1% Triton X-100, 300 mM NaCl, 300 mM sucrose, 1 mM MgCl_2_, 1 mM EGTA, 10 mM PIPES (pH 7.0) supplemented with 2% volume of protease inhibitor cocktail (Nacalai Tesque, 25955–24). Cell lysates were immunoprecipitated using anti-Flag antibody-conjugated agarose beads (ANTI-FLAG M1 Agarose Affinity Gel, A4596, Sigma-Aldrich) and analyzed by Western blotting using anti-Fbxl11 (KDM2A) antibody (Abcam, ab99242), anti-Flag antibody (Sigma), and anti-GFP antibody.

### Recombinant proteins and *in vitro* protein-protein binding assays

The recombinant His-tag HP1γ proteins were expressed in *E.coli* (DH5α) carrying a pCold I vector containing cDNA for HP1γ or HP1γ mutants and isolated using TALON His-tag purification resin (TaKaRa Bio) according to the manufacturer’s instructions. The glutathione S-transferase fusion KDM2A fragments were expressed in *E. coli* (DH5α) using a pGEX-3X vector containing cDNA for KDM2A fragments (785–817 or 785–817, V801E) and isolated using a glutathione-Sepharose column (Amersham Bioscience). The isolated recombinant proteins were dialyzed against a 300 mM NaCl, 50 mM Na-phosphate buffer (pH 7.0).

Recombinant His-tagged HP1γ was incubated with the recombinant KDM2A fragments in 300 mM NaCl, 50 mM Na-phosphate buffer (pH 7.0) at 4° C for 1 h. TALON His-tag purification resin suspended in 1% NP40, 300 mM NaCl, and 50 mM Na-Phosphate buffer (pH 7.0) was added, and further incubated at 4° C for 2 h. The TALON resins were washed with 1% NP40, 300 mM NaCl, and 50 mM Na-phosphate buffer (pH 7.0) three times, and the proteins were extracted by 3% SDS solution containing 100 mM Tris-HCl, pH 6.8, 0.1 M DTT, and 20% glycerol and analyzed by SDS-PAGE.

### siRNA and transfection

Cells were transfected with stealth siRNA using Lipofectamine RNAiMAX (Thermo Fisher Scientific) according to the manufacturer’s instructions. The siRNA oligonucleotide sequence for KDM2A was 5′-GAACCCGAAGAAGAAAGGAUUCGUU-3′, which was described previously [19]. The siRNA oligonucleotide sequences for HP1γ were 5′-CCAAGAGGA UUUGCCAGAGGUCUUG-3′ (#1 oligo) and 5′-GAAAGAAUAAUUGGUGCCACAGACA-3′ (#2 oligo). The control siRNA was control stealth RNA (Stealth RNAi Negative Control Medium GC Duplex, Thermo Fisher).

### Tissues and immunostaining

Routinely processed formalin-fixed and paraffin-embedded specimens from 39 Japanese patients with breast cancer resected from 2011 to 2012 at Kurume University Hospital were used. The breast cancer tissues included papillotubular, solid-tubular, scirrhous, mucinous, and micropapillary carcinomas. Formalin-fixed, paraffin-embedded sections were immunostained with BenchMarkXT (Ventana Medical Systems, Tucson, AZ). An anti-HP1 γ monoclonal antibody was applied at a dilution of 1:1000.

Two pathologists, S.O. and H.Y., who did not know the clinical status of each patient, independently evaluated and interpreted the results of immunostaining using the Allred scoring system [46, 47]. The expression (staining) levels of HP1γ were classified into four categories: tumors that showed the HP1γ staining similar to that in the non-neoplastic epithelial area were classified as 3; tumors that showed intermediate HP1γ staining were classified as 2; tumors that showed weak HP1γ staining were classified as 1; tumors containing no identifiable HP1γ were classified as 0. To estimate the rate of staining, the numbers of positive and negative tumor cells in a field were counted, and the ratio of positive to total cells was expressed as one of six categories: 0/100 as 0, 1/100 as 1, 1/10 as 2, 1/3 as 3, 2/3 as 4, and 1/1 as 5. The ratio of stained cells was added to the strength of the KDM2A staining to produce the HP1γ score. The sections were also characterized by ER, PgR, and HER2 immunostaining as described previously [47], and the results are shown in Table 1. This study was approved by the institutional ethics review board of Kurume University (approval no. 18082).

### RNA extraction and quantitative reverse transcription–polymerase chain reaction (qRT-PCR).

Isolation of total RNA from cells and cDNA synthesis were performed as described previously [19, 20]. The products were diluted to 150 μl with distilled water, and 2.5 μl of the resultant single-strand cDNA was used as the template for qRT-PCR, using Thunderbird SYBR qPCR Mix (Toyobo #QPS-201) with an Mx3000P QPCR system (Agilent Technologies, Santa Clara, CA) according to the manufacturer’s instructions. The values were normalized using the amounts for control mRNA for RNA polymerase II subunit a (Polr2a) [48]. The sets of PCR primers for amplification of the pre-rRNA (a sequence in the 5′ untranslated region 1–155) used were 5′-GCTGACACGCTGTCCTCTG-3′ and 5′-TCGGAC GCGCGAGAGAAC-3′; for KDM2A, the primers used were 5′-TCCCCACACACATTTTGACATC-3′ and 5′-GGGGTGGCTTGAGAGATCCT-3′; for HP1γ, the primers used were 5′ –TGCCAGAGGTCTTG ATCCTGA-3′ and 5′-TCTTTCGCCAGCACCAAG TCT-3′; for Polr2a, the primers used were 5′-ATCTCTCC TGCCATGACACC-3′ and 5′-AGACCAGGCAGGGG AGTAAC-3′ [19].

### Metabolic labelling assay of newly synthesized RNA using 5-ethynyl uridine (EU)

Detection of RNA synthesis temporally was performed by the Click-iT® RNA Imaging Assay kit (Invitrogen, Catalog #C10329), basically according to the manufacturer’s instructions. In brief, after MCF-7 cells were transfected with various siRNAs in 10FCS-RPMI1640 and cultured for three days, cells were replated on glass coverslips. Next day, cells were cultured in the presence or absence of 2 mM 2DG for two hours. Then 5-ethynyl uridine (EU) was added to medium and cells were further cultured for one hour. Cells were fixed with methanol for 30 min at -20° C, and biosynthetic incorporation of EU into newly transcribed *RNA* was detected by Click-iT reaction buffer.

Cells expressing the wild-type KDM2A or mutant KDM2A (V801E) under the tet-on system cultured in 10 FCS-RPMI1640 were transfected with siRNA for KDM2A and incubated in the presence of 0.5 μg/ml Dox. After two days, cells were replated on coverslips and cultured in 10FCS-RPMI1640 in the presence of 0.5 μg/ml Dox for two days. Cells were further incubated in the presence or absence of 2 mM 2DG for two hours and newly synthesized RNA was detected using EU, as described above.

To detect the effects of a knockdown of HP1γ or KDM2A on rRNA transcription in the presence of 2DG in MDA-MB-231 cells, cells were transfected with various siRNAs in 10FCS-DMEM and cultured for two days, and replated on glass coverslips in 10FCS-DMEM. Next day, cells were incubated in the presence or absence of 3 mM 2DG for two hours. Then EU was added to medium and biosynthetic incorporation of EU into newly transcribed *RNA* was detected, as described above.

## SUPPLEMENTARY MATERIALS



## Abbreviations

AMPK: AMP-dependent protein kinase
CD: chromodomain
CSD: chromoshadow domain
CxxC-zf: CxxC-zinc finger
DAPI: 4’,6-diamidino-2-phenylindole
2DG: 2-deoxyglucose
DMEM: Dulbecco’s modified Eagle’s medium
EU: 5-ethynyl uridine
GFP: green fluorescence protein
HP1: heterochromatin protein 1
NoLS: nucleolar localization sequence
Pol I: RNA polymerase I
rDNA: ribosome RNA genes
rRNA: ribosome RNA
TNBC: triple negative breast cancer
