# Supplementary material for: KDM2A-dependent reduction of rRNA transcription on glucose starvation requires HP1 in cells, including triple-negative breast cancer cells
Source: Oncotarget. 2019 Jul 30;10(46):4743–60. doi: 10.18632/oncotarget.27092 (PMC6677663; doi:10.18632/oncotarget.27092)
Supplement: Supplementary file 1 [file oncotarget-10-4743-s001.pdf]

KDM2A-dependent reduction of rRNA transcription on glucose starvation requires HP1 in cells, including triple-negative breast cancer cells

SUPPLEMENTARY MATERIALS

REFERENCES

1. Scott MS, Troshin PV, Barton GJ. NoD: a Nucleolar localization sequence detector for eukaryotic and viral proteins. BMC Bioinformatics. 2011; 12:317. https://doi.org/10.1186/1471-2105-12-317. [PubMed]
2. Tsukada Y, Zhang Y. Purification of histone demethylases from HeLa cells. Methods. 2006; 40: 318–326. https://doi.org/10.1016/j.ymeth.2006.06.024. [PubMed]
3. Lechner MS, Schultz DC, Negorev D, Maul GG, Rauscher FJ. The mammalian heterochromatin protein 1 binds diverse nuclear proteins through a common motif that targets the chromoshadow domain. Biochem Biophys Res Commun. 2005; 331:929–937. https://doi.org/10.1016/j.bbrc.2005.04.016. [PubMed]
4. Thiru A, Nietlispach D, Mott HR, Okuwaki M, Lyon D, Nielsen PR, Hirshberg M, Verreault A, Murzina NV, Laue ED. Structural basis of HP1/PXVXL motif peptide interactions and HP1 localisation to heterochromatin. EMBO J. 2004; 23:489–499. https://doi.org/10.1038/sj.emboj.7600088. [PubMed]
5. Nozawa RS, Nagao K, Masuda HT, Iwasaki O, Hirota T, Nozaki N, Kimura H, Obuse C. Human POGZ modulates dissociation of HP1alpha from mitotic chromosome arms through Aurora B activation. Nat Cell Biol. 2010; 12:719–727. https://doi.org/10.1038/ncb2075. [PubMed]

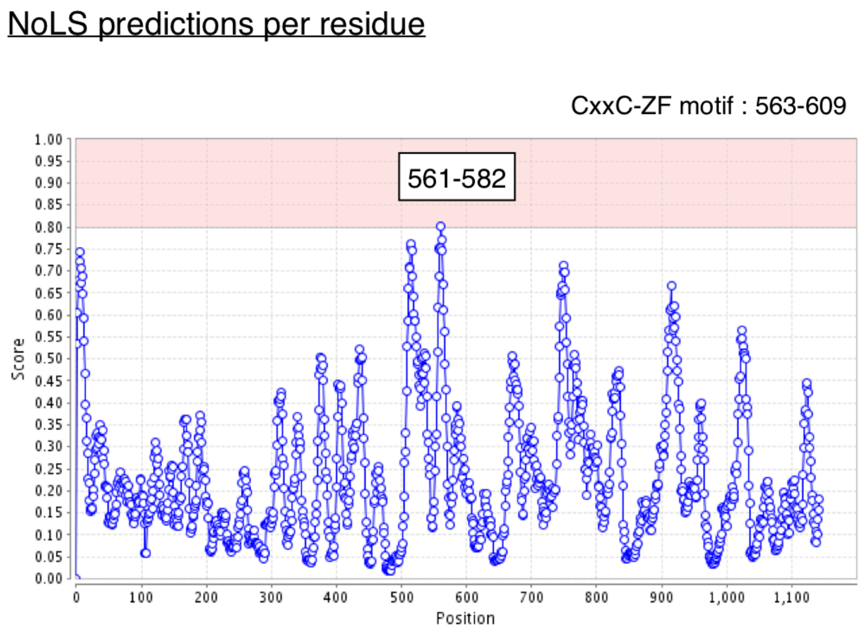

Supplementary Figure 1: The amino acid sequence of human KDM2A was subjected to a nucleolar localization sequence detector for eukaryotic and viral proteins [1]. One region, amino acids 561–582 of KDM2A, which was overlapped with a CxxC-ZF motif (563-609), was suggested to be a nucleolar localization sequence.

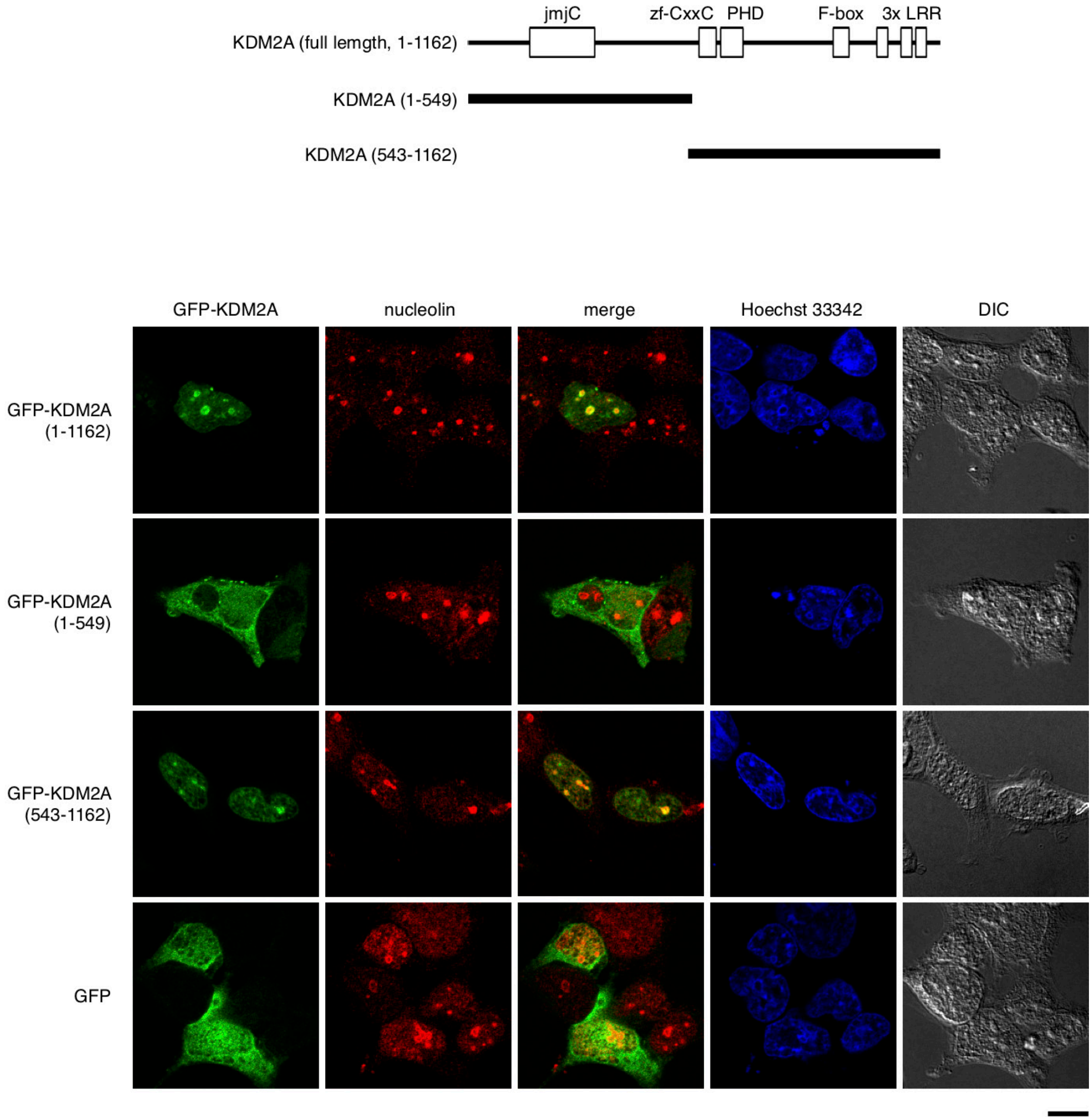

Supplementary Figure 2: KDM2A and its deletion mutants. Functional domains were identified using the SMART program [2]. Schematic diagrams of the different KDM2A deletion mutants, which were fused with GFP on the C-terminal side of KDM2A, are shown. The GFP fusion proteins were expressed in MCF-7 cells, and the subcellular localizations of GFP (Green), nucleolin (Red), and nuclei (Blue) were observed. Scale bar corresponds to 10 μm.

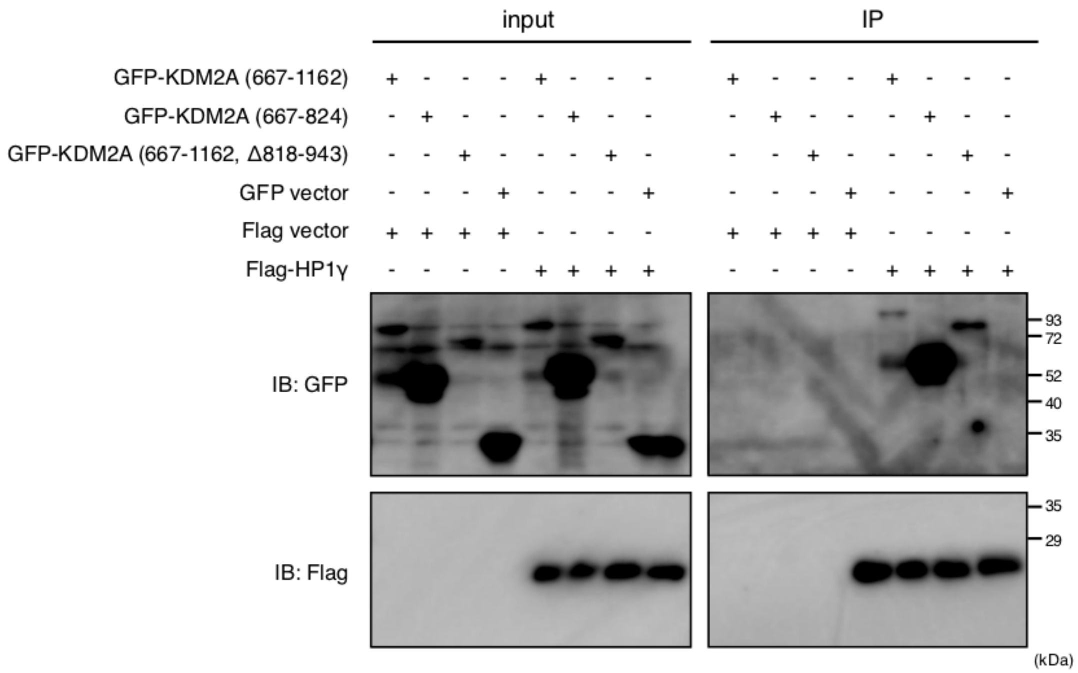

Supplementary Figure 3: An expression vector encoding Flag-HP1γ or the empty vector was cotransfected with a pEGFP vector encoding KDM2A (amino acids 667–1162), KDM2A (amino acids 667-824), or KDM2A (amino acids 667–1162, Δ818–924) to 293T cells. Cell lysates were immunoprecipitated by an anti-Flag antibody, and analyzed by Western blotting using an anti-GFP or anti-Flag antibody. One tenth of input samples were also analyzed.

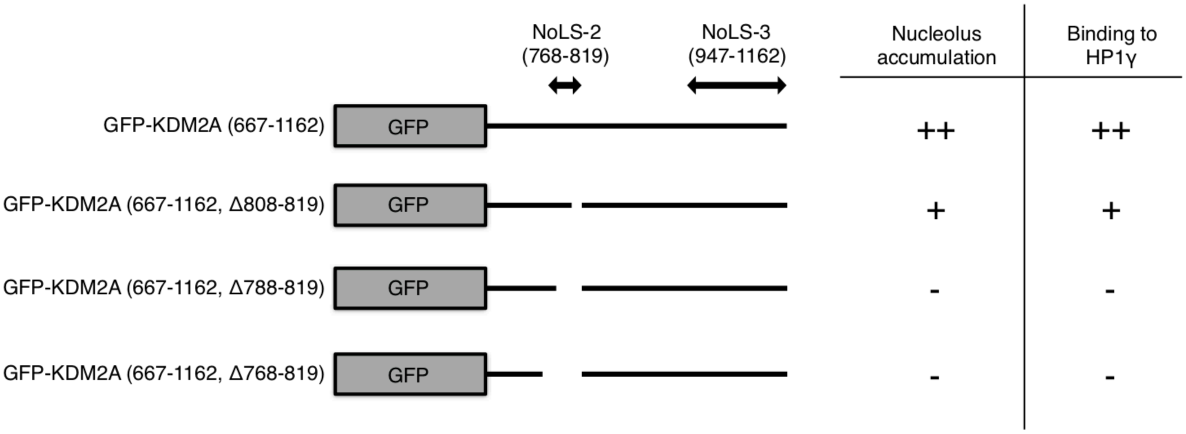

Supplementary Figure 4: Summary of the results in Figure 2. Schematic diagrams of the KDM2A fragments fused with GFP. The KDM2A fragment ranging from 667–1162 (C-terminal end) or the fragments with a partially deleted NoLS-2 region was fused with GFP. NoLS-2 and NoLS-3 regions are shown. The results of the efficiencies of nucleolar accumulation (Figure 2C) and HP1γ-binding (Figure 2B) are summarized on the right..

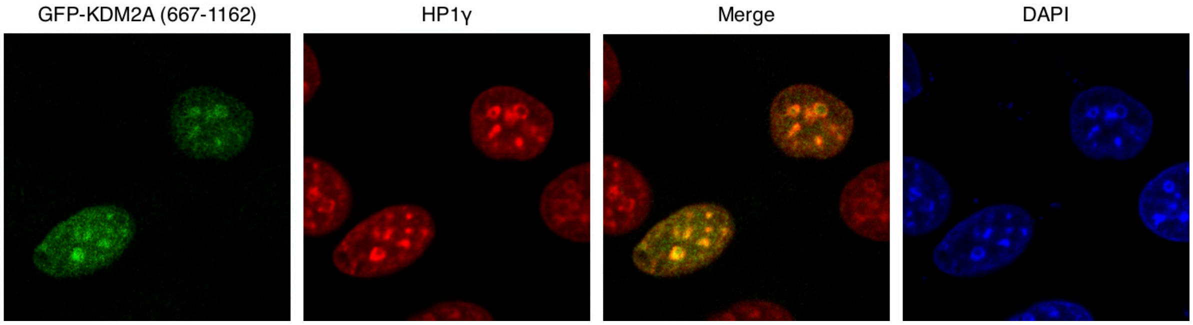

Supplementary Figure 5: Localization of GFP fusion KDM2A (amino acids 667–1162) and endogenous HP1γ. GFP fusion KDM2A (amino acids 667–1162) were expressed in MCF-7 cells, and HP1γ was immunostained by an anti-HP1γ antibody. Signals for GFP (Green), HP1γ (Red), and nuclei (Blue) were detected. Scale bar corresponds to 10 µm.

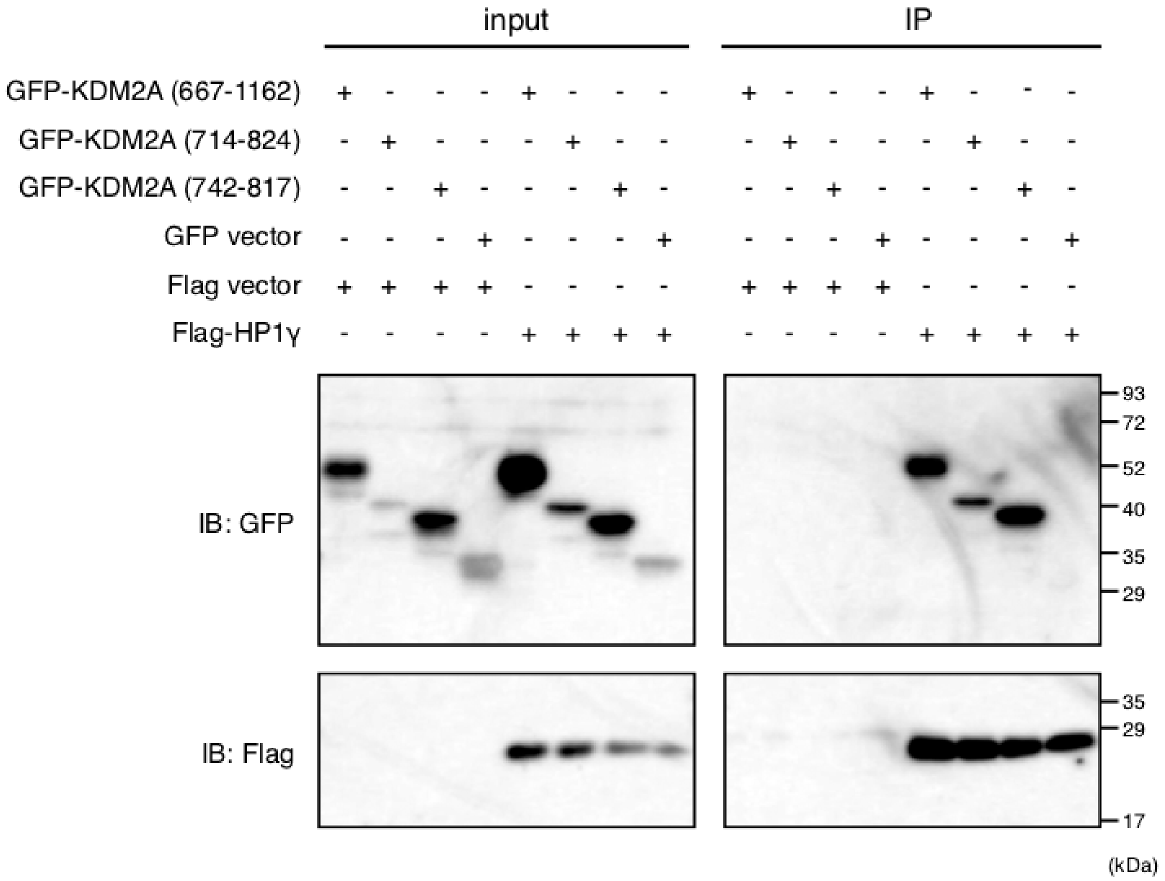

Supplementary Figure 6: An expression vector encoding Flag-HP1γ or the empty vector was cotransfected with pEGFP vectors encoding KDM2A (amino acids 667–1162), KDM2A (amino acids 714–824), or KDM2A (amino acids 742–812) to 293T cells. Cell lysates were immunoprecipitated by an anti-Flag antibody, and analyzed by Western blotting using anti-Flag and anti-GFP antibodies. One tenth of input samples were also analyzed.

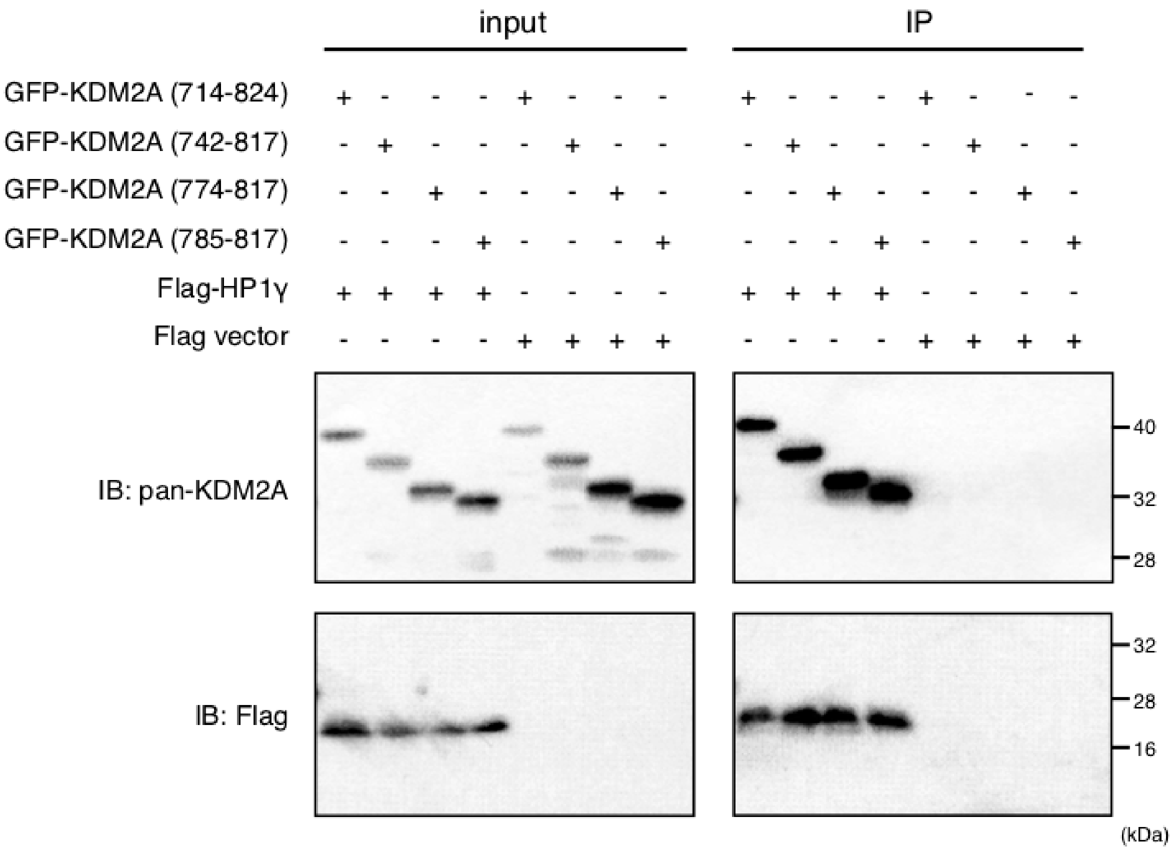

Supplementary Figure 7: An expression vector encoding Flag-HP1γ or the empty vector was co-transfected with a pEGFP vector encoding KDM2A (amino acids 714–824), KDM2A (amino acids 742–817), KDM2A (amino acids 774–817), or KDM2A (amino acids 785–817) to 293T cells. Cell lysates were immunoprecipitated by an anti-Flag antibody, and analyzed by Western blotting using an anti-GFP or anti-Flag antibody. One tenth of input samples were also analyzed.

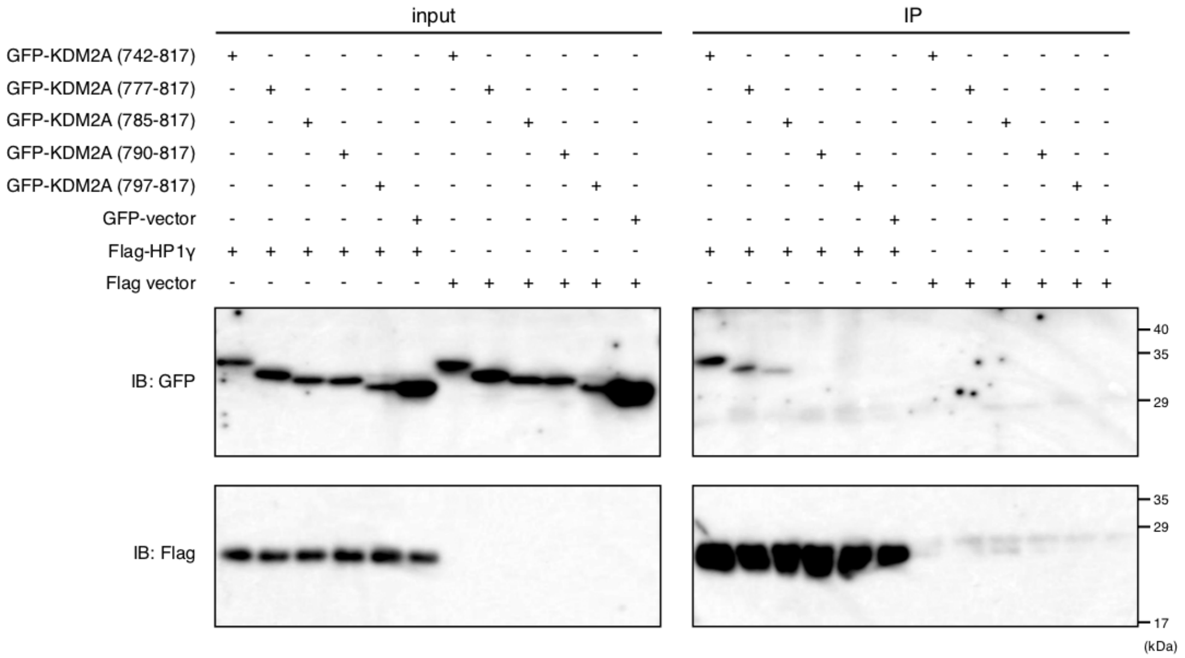

Supplementary Figure 8: An expression vector encoding Flag-HP1γ or the empty vector was cotransfected with a pEGFP vector encoding KDM2A (amino acids 742–817), KDM2A (amino acids 777–817), KDM2A (amino acids 785–817), KDM2A (amino acids 790–817), or KDM2A (amino acids 797–817) to 293T cells. Cell lysates were immunoprecipitated by an anti-Flag antibody and analyzed by Western blotting using an anti-GFP or anti-Flag antibody. One tenth of input samples were also analyzed.

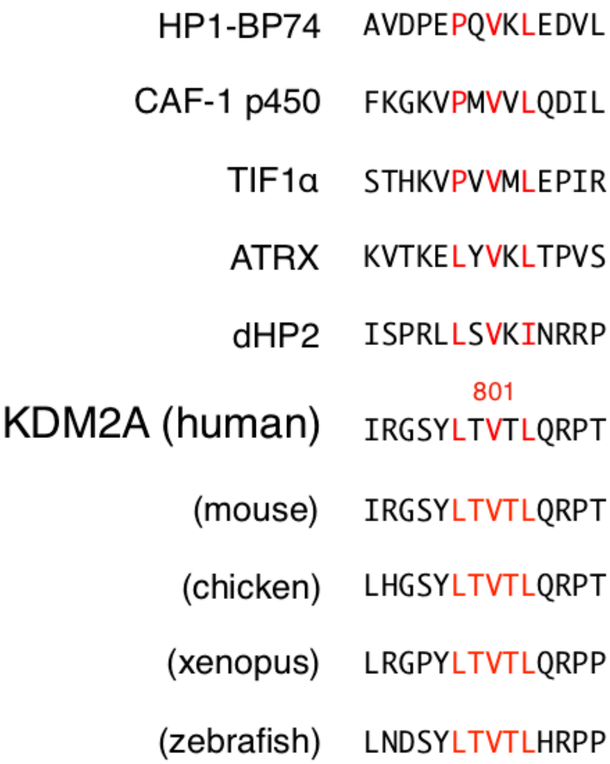

Supplementary Figure 9: The chromoshadow domain (CSD) of HP1 binds many HP1-interacting proteins, including HP1-BP74, CAF-1 p450, and TIF1a [3-5] through a PxVx(M/L/V) motif. In addition, an LxVxL motif was also rarely reported to bind HP1 protein. KDM2A has the LxVxL motif within an HP1γ-binding stretch (amino acids 785–810). This motif is conserved among KDM2A in vertebrates.

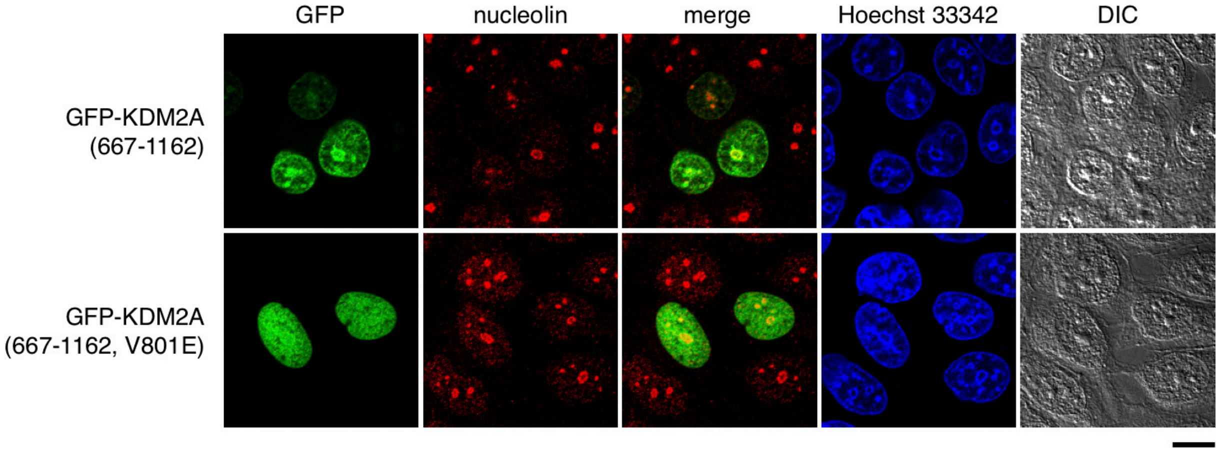

Supplementary Figure 10: GFP fusion KDM2A (amino acids 667–1162) or KDM2A (amino acids 667–1162, V801E) were expressed in MCF-7 cells. The localizations of GFP (Green), nucleolin (Red), and nuclei (Blue) were observed. Scale bar corresponds to 10 μm.

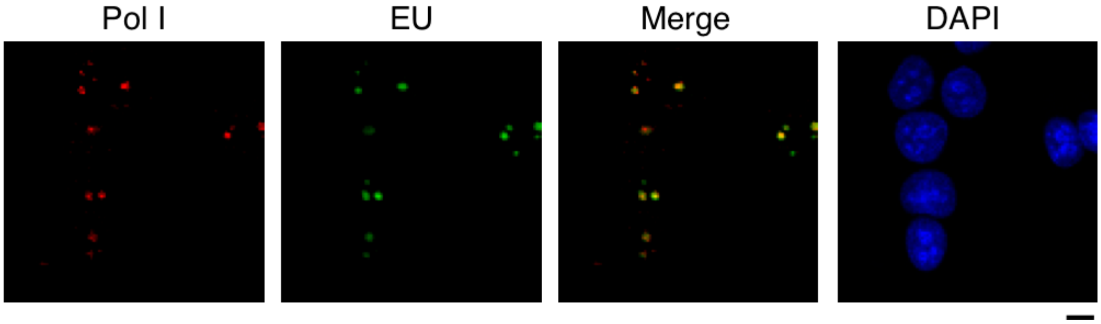

Supplementary Figure 11: MCF-7 cells replated on glass coverslips in 10FCS-RPMI1640 were cultured in the presence of 5-ethynyl uridine (EU) for one hour. Cells were fixed with methanol for 30 min at –20° C. Biosynthetic incorporation of EU was detected by Click-iT reaction buffer of Click-iT® RNA Imaging Assay kit (Invitrogen, Catalog #C10329), according to the manufacturer's instructions. RNA polymerase I in cells labeled with EU was detected by anti-mouse RNA polymerase I antibody and then Alexa 568-labeled anti-mouse antibody and DAPI. Cells were observed thorough fluorescent microscopy. The signals of EU were overlapped with the signals for Pol I. Scale bar corresponds to 10 µm.
